# Burnout syndrome in healthcare professionals almost two years after the declaration of the Covid-19 pandemic

**DOI:** 10.1192/j.eurpsy.2022.1357

**Published:** 2022-09-01

**Authors:** M. Solis, A. Jurado Arevalo, E. Blánquez Garcia

**Affiliations:** Universitary Hospital of Jaén, Psychiatric, Jaén, Spain

**Keywords:** Healtcare professionals, burnout, mental health, Covid-19

## Abstract

**Introduction:**

The coronavirus disease 2019 (COVID-19) pandemic has caused major sanitary crisis worldwide. Frontline healthcare workers face many difficulties, such as: direct exposure to patients with high viral load, physical exhaustion, reorganization of workspaces, face the unusually high number of deaths among patients, colleagues or relatives and ethical issues in a tense health system.

**Objectives:**

Provide up-to-date information of Burnout syndrome associated with exposure of healthcare workers to the COVID-19 pandemic, after almost 20 months of the declaration of pandemic by the World Health Organization.

**Methods:**

A cross-sectional study was carried out that included 84 healthcare workers from Spain in October 2021, through an anonymous, voluntary and multiple response type online survey which included questions about sociodemographic aspects and the Maslach burnout inventory

**Results:**

62% were doctors and 29% were nurses. 70% work on the front line of Covid-19. 38% report not having been able to enjoy their vacations when they wanted. 8% admit to having had suicidal ideas. Almost 52% admit low personal fulfillment, 38.6% admit a high depersonalization count, and 45% report high emotional exhaustion. Of the total sample, 17 respondents have burnout syndrome.

**Conclusions:**

It is necessary create strategies to promote mental well-being in health professionals exposed to COVID-19 after 20 months of active work. Protecting and identifying health care professionals who could be at high risk for developing a mental health pathology or detecting Burnout syndrome in them should be the priority of public health post pandemic.

**Disclosure:**

No significant relationships.

